# Reactive Oxygen Species as Regulators of MDSC-Mediated Immune Suppression

**DOI:** 10.3389/fimmu.2018.02499

**Published:** 2018-10-30

**Authors:** Kim Ohl, Klaus Tenbrock

**Affiliations:** Department of Pediatrics, Medical Faculty, RWTH Aachen, Aachen, Germany

**Keywords:** ROS, MDSC, Nrf2, redox regulation, metabolism

## Abstract

Reactive oxygen species (ROS) molecules are implicated in signal transduction pathways and thereby control a range of biological activities. Immune cells are constantly confronted with ROS molecules under both physiologic and pathogenic conditions. Myeloid-derived suppressor cells (MDSCs) are immunosuppressive, immature myeloid cells and serve as major regulators of pathogenic and inflammatory immune responses. In addition to their own release of ROS, MDSCs often arise in oxidative-stress prone environments such as in tumors or during inflammation and infection. This evidently close relationship between MDSCs and ROS prompted us to summarize what is currently known about ROS signaling within MDSCs and to elucidate how MDSCs use ROS to modulate other immune cells. ROS not only activate anti-oxidative pathways but also induce transcriptional programs that regulate the fate and function of MDSCs. Furthermore, MDSCs release ROS molecules as part of a major mechanism to suppress T cell responses. Targeting redox-regulation of MDSCs thus presents a promising approach to cancer therapy and the role of redox-signaling in MDSCs in other disease states such as infection, inflammation and autoimmunity would appear to be well worth investigating.

## Introduction

Reactive oxygen species (ROS) appear to have harmful as well as beneficial effects(1, 2). Their harmful effects include oxidation-induced damage to cellular contents, such as lipids, proteins, carbohydrates and nucleic acids, which subsequently induce cell pathologies and cell death. Damaged and oxidized molecules contribute to a number of alterations including atherosclerosis, neurodegenerative diseases and aging. Beyond this, ROS molecules are implicated in signal transduction pathways and redox-dependent regulations controlling a range of biological activities. In this regard, it is interesting to examine myeloid-derived suppressor cells (MDSCs). These heterogenic myeloid cells are controlled by ROS but they also use ROS to fulfill suppressive functions. Pathological conditions such as chronic inflammation, infection and cancer, induce MDSCs, which consist of a heterogeneous population of immature myeloid cells (3, 4). A hallmark of these immunosuppressive cells is their capability to suppress T cell responses, which contributes to cancer immune evasion on the one hand but suppression of exaggerated T cell responses during inflammation on the other. In mice, MDSCs are broadly characterized by the surface expression of CD11b and Gr-1 and are further grouped into monocytic (CD11b^+^Ly6C^high^Ly6G^−^) and polymorphonuclear (CD11b^+^Ly6C^low^Ly6G^+^) MDSCs (5). The polymorphonuclear (PMN-MDSC) subset displays increased STAT3 and NADPH oxidase (Nox) activity, which results in high release of ROS but low NO release. The monocytic subset (M-MDSC) express high levels of STAT1 and iNOS and enhanced level of NO but show low ROS production. Both of them express arginase 1 (3). Although ROS have toxic effects on most cells, MDSCs survive despite elevated levels and continuous production of ROS (6). ROS production is not only central to the immunosuppressive properties of MDSCs but also seems to maintain them in an undifferentiated state. Furthermore, steady-state production of ROS by MDSCs is upregulated in a variety of murine tumor models and in human cancer, and also after activation in inflammatory and autoimmune conditions (4, 7). In addition to their own ROS release, MDSCs often arise in oxidative-stress prone environments such as in tumors or during inflammation and infection. This evidently close relationship between MDSCs and ROS prompted us to summarize what is currently known about ROS signaling in MDSCs itself and to elucidate how MDSCs use ROS to modulate other immune cells.

## Main text

### Regulation of MDSCs by ROS

A state of “oxidative stress” describes a situation where high levels of ROS -derived from cellular metabolism, toxic insults, or oxidative burst- outbalance the anti-oxidative system (8). This breakdown of cellular homeostasis results from mitochondrial dysfunction or increased metabolic activity, oncogene activity or infiltrating immune cells (8) and induces damage to lipids, proteins, carbohydrates and nucleic acids and can even lead to cell death (9). Excessive production of ROS molecules is associated with several inflammatory and pathologic conditions. For example, oxidative stress within the intestinal epithelium is thought to be involved in the pathogenesis of intestinal inflammation (10) and oxidative stress is also associated with neurodegenerative diseases (8). Furthermore, elevated rates of ROS can be observed in almost all cancers and are involved in tumor metastasis (11). On the other hand, emerging evidence suggests that ROS molecules serve as signaling intermediates that play central roles in several molecular pathways and also serve as central mediators of immune cells (12). Low levels of ROS are continuously generated under healthy cellular conditions, and are neutralized by the endogenous antioxidant machinery that is regulated by nuclear factor (erythroid-derived 2)-like 2 (Nrf2). Nrf2 is retained and degraded in the cytosol by Kelch ECH associating protein 1 (Keap1) under basal conditions (13). Cellular stimuli such as oxidative stress lead to conformational changes in Keap1, which are followed by the release of Nrf2 from Keap1. Afterwards, Nrf2 translocates into the nucleus, where transactivation of genes containing an antioxidant response element (ARE) in their promoter regions takes place (14). Thereby, Nrf2 up-regulates phase II detoxifying enzymes and antioxidant proteins. These processes play a vital role in maintaining cellular homeostasis and are of major relevance upon exposure of cells to chemical or oxidative stress and inflammation. Particulary enzymes mediating gluathione (GSH) synthesis, the thioredoxin enzyme system, and detoxifying enzymes such as heme oxygenases, or NAD(P)H: quinone oxidoreductase 1 are part of the Nrf2 induced enzymatic machinery.

The most prevalent intracellular sources of ROS are mitochondria and NADPH oxidases (Nox) but beyond this, the ER and also the peroxisome (organelle that metabolizes long chain fatty acids) generate ROS molecules. Nox-mediated release of ROS induces the so-called oxidative burst and eliminates invading microorganisms (15). The relevance of Nox-derived ROS in host immunity is best demonstrated by the disease pattern of chronic granulomatous disease (GCD), which is caused by NOX2 defects, and results in hypersensitivity to common infections and accumulation of bacteria-containing phagocytes with subsequent granulome development (15, 16).

Mitochondrial ROS are central regulators of the innate immune system. They are indispensable for Toll-like receptor (TLR)-initiated pathways (17). In detail TLR1, TLR2, and TLR4 signaling leads to recruitment of mitochondria to phagosomes and enhances ROS production in macrophages, indicating that mitochondrial ROS form an important component of antibacterial responses and are necessary for activation of NLRP3 inflammasome (18). In addition to this, mitochondrial ROS are involved in NLRP3 activation. Accumulation of damaged ROS-generating mitochondria leads to NLRP3 activation (19), and increased levels of mitochondrial ROS resulting from NLRP3 activation serve as a feedback mechanism to sustain activation (20).

**Figure 1:**
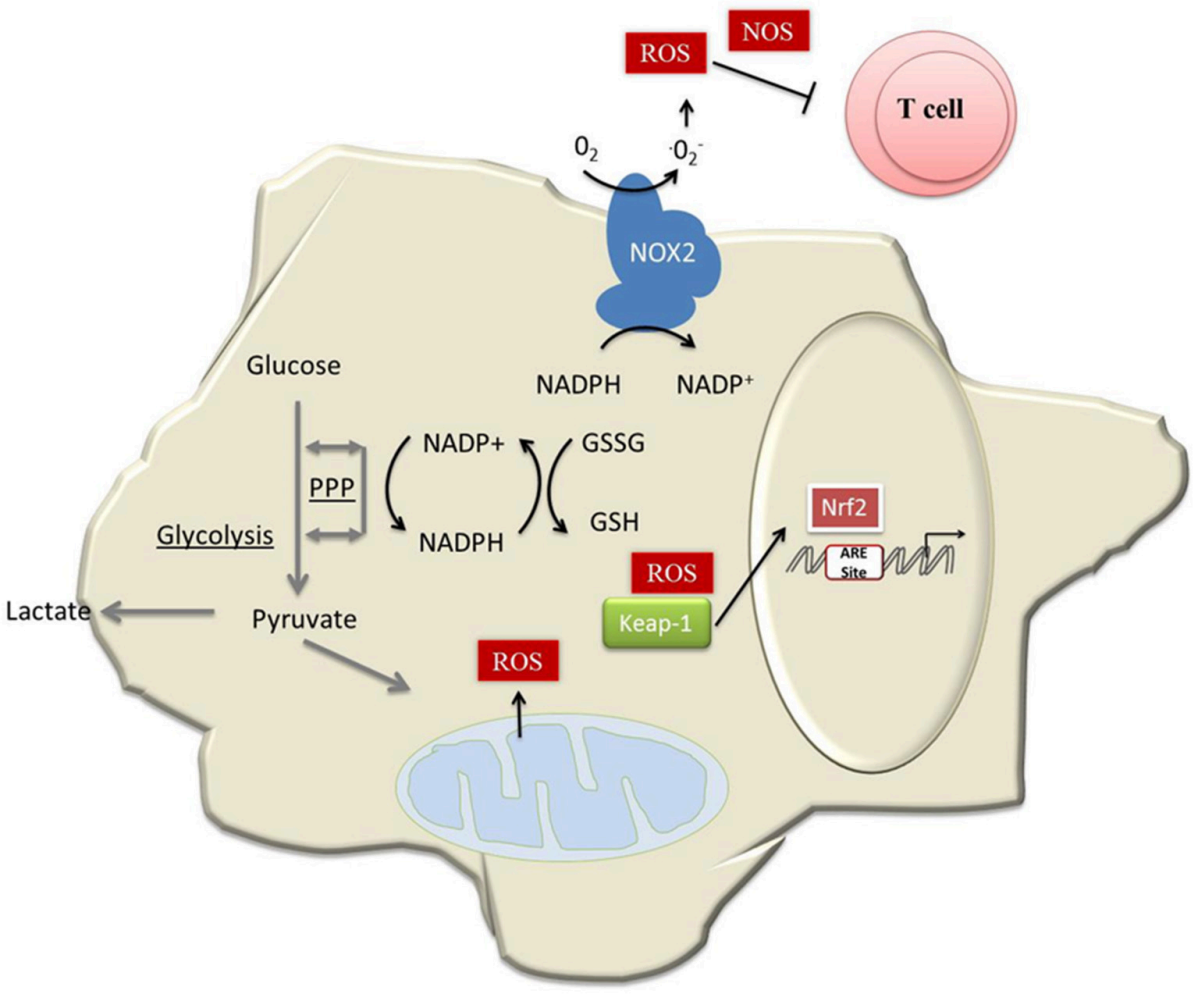
Model of how MDSCs maintain redox homeostasis. Activated MDSCs produce high amounts of ROS molecules by the action of NOX2 (4). This, in addition to mitochondrial ROS and ROS derived from cancer cells, compromises redox homeostasis in MDSCs and most likely induces apoptosis in the absence of Nrf2 (21). Another consequence of Nrf2 activation, besides expression of antioxidative genes, is a metabolic reprogramming of MDSCs. This leads to enhanced expression of the PPP (22), which provides GSH. GSH not only serves as a major antioxidant, but is essential for MDSC differentiation (22). In addition, MDSCs counteract OXPHOS-derived ROS by upregulation of glycolysis (23).

Furthermore, mitochondrial ROS and ROS derived from other sources and cellular metabolism are intimately linked. Oxidative phosphorylation (OXPHOS) is a major cellular source of ROS and requires adequate availability of antioxidants to prevent apoptosis. One advantage of glycolysis over OXPHOS lies therefore in a better maintenance of the redox balance. Lian et al. recently observed that MDSCs counteract OXPHOS-derived ROS by upregulation of glycolysis, thereby protecting MDSCs from apoptosis (Figure 1) (23). We observed high OXPHOS in MDSCs of mice with a constitutive Nrf2 activation and subsequently low levels of intracellular ROS (22). The constitutive activation and availability of antioxidant enzymes regulated by Nrf2 activation in these cells might be a central mechanism enabling the cells to increase mitochondrial ATP production by simultaneously counteracting subsequent high ROS levels. High oxygen consumption rate (OCR) levels were associated with a highly suppressive and tolerizing phenotype. Recent studies have shown that aerobic glycolysis constitutes the metabolic basis for trained immunity (24). The metabolism of tolerant myeloid cells, particularly of MDSCs, is less clearly understood and was one focus of our study (22). It is generally assumed that naïve or tolerant cells primarily use OXPHOS as an energy source, but activated cells, e.g., after LPS stimulation, undergo a shift toward aerobic glycolysis (25). However, metabolic characteristics of MDSCs seem to differ within this quite heterogeneous cell population and might also depend on disease context. In comparison to splenic MDSCs, tumor-infiltrating MDSCs enhance fatty acid oxidation (26). However, rapamycin, which specifically inhibis mTOR, reduces M-MDSC in mice with allografts or tumors (27). Flux of glucose down the pentose phosphate pathway (PPP) is essential for redox buffering as PPP produces NADPH. This is required to maintain GSH, the most important cellular antioxidant, in the reduced state (Figure 1). Again, we observed a high expression of PPP enzymes in Nrf2-activated MDSCs, which suggests that Nrf2 is critically involved in redox and in the metabolic signaling of MDSCs, and acts either by mediating ROS signaling or possibly also by targeting other genes (22).

Redox signaling is moreover involved in several signal transduction pathways. In most cases cystein (Cys) residues serve as redox-dependent switches and the oxidation/reduction of specific amino acids, that bear reactive Cys residues, induces activation, or inactivation of target proteins such as phosphatases. Moreover, ROS modulate antioxidant enzymes that not only serve as scavengers but also transduce redox-dependent signals (28). GSH is not only the most important antioxidant in cells in general but also mediates specific effects in immune cells. For instance, GSH is involved in reprogramming of effector T cells during inflammation (29). With regard to MDSCs, increased levels of GSH are especially important for MDSC differentiation (30). Probably, GSH affects MDSCs differentiation by neutralization of ROS but other direct effects of GSH on MDSCs are conceivable.

ROS molecules are essential for maintainance of MDSCs in their undifferentiated state. Scavenging of H_2_O_2_ with catalase induces differentiation of immature myeloid cells into macrophages in mice bearing tumors (31), while in the absence of Nox activity, MDSCs differentiate into macrophages and DCs in tumor-bearing mice (4). Increased levels of endogenous H_2_0_2_ might thereby present a mechanism by which tumors prevent the differentiation of MDSCs. The precise molecular mechanism maintaining MDSCs in their undifferentiated state in the presence of ROS remains to be elucidated.

### Regulation of cellular immune responses by MDSC-derived ROS

Release of ROS molecules is one of the major mechanisms that MDSCs use to suppress T cells in mice and humans (4, 32, 33). Administration of ROS inhibitors was found to counteract the suppressive effect of human MDSCs on T cells (34). And, at least in tumor-bearing mice, suppression of T cells is dependent on NOX2 activity (4). Superoxide released by MDSCs rapidly reacts with a large number of molecules e.g., H_2_O_2_, hydroxyl radical, hypochlorous acid, and peroxynitrite to form ROS, which then damage proteins, lipids, and nucleic acids, enhance inflammation and promote apoptosis. ROS are even thought to enable Ag-specific suppression of T cell responses by MDSCs. Nagaraj et al. showed that MDSC-derived ROS molecules and peroxynitrite, which is the product of the reaction of ROS with NO, modify TCR and CD8 molecules. Through these modifications, CD8^+^ T cells lose their ability to bind phosphorylated MHC and induce antigen-specific tolerance of peripheral CD8^+^ T cells (35).

H_2_0_2_, formed from MDSC-derived superoxide, decreases T cellular CD3ζ expression, thereby limits the ability of the T cells to become activated (36) and reduces their expression of IFN-γ (4).

While MDSCs suppress effector T cells, they induce the expansion of regulatory T cells (T_regs_) in cancer, and also in inflammatory conditions (37–42). Induction of T_regs_ is therefore one important mechanism of MDSC-mediated T cell inhibition. The role of ROS molecules in the interaction of MDSCs and T_regs_ is not clear. The induction of T_reg_ cells by macrophages involves production of ROS and therefore ROS deficiency might lead to reduced T_reg_ induction and might aggravate T-cell suppression (43). In addition, T_regs_, are less susceptible to oxidative stress-induced cell death compared to other T cell populations (44). This is most likely caused by a greater secretion of redox proteins such as thioredoxin (44) or hemeoxygenase 1 (45). In addition to this, human T_regs_ have been shown to express high levels of cell surface thiols, that are important reducing agents, and facilitate enhanced intracellular anti-oxidative abilities (44). Nevertheless, a recent study claims that T_reg_ cells are less resistant to oxidative stress in the tumor microenvironment compared to conventional T cells and even undergo ROS-induced apoptosis due to a weak Nrf2-associated antioxidant system (46). These apoptotic T_reg_ cells suppress antitumor T cell immunity even more efficiently via the adenosine and A_2A_ pathways. As a consequence, T_regs_ or at least T_reg_-mediated suppression seems to benefit from oxidative stress conditions and might therefore contribute to MDSC-mediated immune suppression.

Beyond direct effects on T cells, ROS molecules also indirectly modulate T cell responses. Peroxynitrite indirectly hinders T cells activation by modifying the antigen presenting structure on tumor cells. To this end, peroxynitrite reduces the binding of antigens to tumor cell-associated MHC and thereby generates tumor cells that are resistant to antigen-specific cytotoxic T cell responses (47). Furthermore reactive nitrogen species induce posttranslational modifications of T cell chemokines and thereby hinder antigen-specific T cells invasion of tumors (48).

Furthermore, not only T cell responses are targets of ROS mediated suppression by MDSCs. PMN-MDSCs also suppress NK cell responses to adenoviral vectors and to vaccinia virus infection by ROS release (49, 50). In addition, MDSCs also suppress NK cell toxicity in tumor bearing mice and might critically contribute to the attenuated NK cell activity and cytotoxicity in tumors (51), however the exact mechanism and involvement of ROS are not fully determined.

Recent research demonstrates that MDSCs also negatively regulate B cell- mediated immune responses using ROS. In a murine AIDS model (LP-BM5 reotroviral infection) M-MDSC suppressed B cell responses at least in part by ROS mediated suppression (52, 53). Astudy with human PMN-MDSCs demonstrates that MDSCs suppress B cell proliferation and antibody production in a cell contact manner by means of arginase, NO and ROS (54).

## Redox-dependent transcriptional reprogramming of MDSCs in cancer and inflammation

It is of note that HIF-1α and Nrf2, which are both involved in redox-signaling and oxidative stress responses, emerge as critical regulators of MDSCs. Beyond redox regulation; both factors control other mechanisms and thereby regulate MDSC fate and function.

A critical role of HIF-1α signaling in MDSCs is described in murine cancer models, such as hepatocellular carcinoma (HCC) (21, 55, 56) and in patients with non-small cell lung cancer (57). Interestingly, HIF-1α controls the manner of MDSC-mediated suppression, depending on the hypoxic state of the environment. The dominant mechanism in peripheral lymphoid organs is mediated by ROS and results in antigen-specific T cell non-responsiveness. However, within the hypoxic tumor microenvironment, MDSCs bearing the same phenotype and morphology revealed low levels of ROS levels but significantly enhanced NO production as well as arginase activity and thereby suppressed T cells (21). Several mechanisms have been analyzed by which HIF-1α regulates the fate and function of MDSCs in a hypoxic tumor environment. Some of these studies come to contradictory conclusions, possibly due to the use of different tumor models or the heterogeneity of MDSC populations. Liu et al. showed that lineage differentiation of MDSCs to M1 cells requires glycolytic activity induced by mTOR- and HIF-1α, as brought about by SIRT1 in tumors (56), while Cocl2 (an HIF-1α activator) effectively promotes M1-MDSC differentiation, and potentiates tumor-killing and glycolytic activities. On the other hand, HIF-1α was found to upregulate PD-L1 on MDSCs and induce miR-210, both of which enhance MDSC-mediated T cell suppression (58, 59). In conclusion, these studies reveal that by regulating several pathways including metabolic reactions and miRNA expression, HIF-1α critically regulates the function and maintenance of MDSCs within the hypoxic tumor environment.

Nrf2 is involved in the regulation of various pathways in MDSCs as well. Through an analysis of Nrf2-deficient mice in mammary carcinoma and colon carcinoma models, Beury et al. initially showed that Nrf2 regulates numbers and function of MDSCs (6). Nrf2 deficient mice had increased survival rates and reduced tumor progression with reduced numbers of MDSCs and MDSCs from Nrf2-deficient mice had a reduced suppressive capacity and, surprisingly, a reduced H_2_0_2_ production. Intracellular oxidative stress and apoptosis were enhanced in the absence of Nrf2. However, myeloid-lineage specific Nrf2 deficiency enhances lung metastasis and has been shown to lead to an aberrant ROS accumulation in myeloid cells (60). Nrf2 is known to play dual roles in cancer prevention and progression, which depends on the cellular context and environment (61). However, the exact mechanisms involved remain to be elucidated. It is also not clear whether Nrf2 expression—like HIF-1α expression—is different in peripheral lymphoid organs and tumor MDSCs and whether it might therefore also influence local MDSC maintenance. We observed spontaneously enhanced numbers of MDSCs in mice with a constitutive activation of Nrf2 with intact suppressive functions *in vitro*. This was also found in a transfer colitis model and in a sepsis model *in vivo* (22). MDSCs with constitutive Nrf2 activation displayed low levels of intracellular ROS, but a high metabolic activity and high proliferation rates. This suggests that, beyond its anti-oxidative action, Nrf2 has several other effects that need to be taken into account and might contribute to a context-dependent regulation of MDSCs.

## Conclusion

ROS signaling is without doubt a central mediator of MDSC function and fate. Furthermore, beyond their role in MDSC-mediated immune-suppression, ROS molecules are intrinsically involved in activation of transcription factors such as Nrf2 and HIF-1α, which can induce transcriptional and metabolic reprogramming of MDSCs and influence their differentiation and maintenance. Compounds that target ROS in MDSCs to enhance the effects of cancer immune therapy are promising therapeutic options. The synthetic triterpenoid C-28 methyl ester of 2-cyano-3,12-dioxooleana-1,9,-dien-28-oic acid (CDDO-Me, also referred to as bardoxolone methyl, RTA402, TP-155 and NSC713200) is a potent Nrf2 activator and has been found to reduce MDSC production of ROS and tumor growth in mouse tumor models (62). CDDO-Me shows a promising anticancer effect in a phase I trial (63). In addition, systemic treatment with all-trans-retinoic acid (ATRA) promotes maturation of human MDSCs and reverses their immune suppressor function. Accumulation of GSH in MDSCs by ATRA decreases levels of ROS and induces MDSC differentiation into mature myeloid cells (30, 64). Until now, most studies have focused on cancer models and suggest that inhibition of ROS production in MDSCs helps to enhance anti-tumor immune responses. Beyond their pathogenic role in cancer, expansion and activation of MDSCs also occurs in autoimmunity, infection and chronic inflammation, conditions that are associated with oxidative stress and hypoxic states (10, 65, 66). Thus, redox-signaling in MDSCs might be a promising therapeutic target in these diseases as well. However, the role of MDSCs here seems to be less clear here, and both positive and negative roles of MDSCs have been revealed with regard to progression of autoimmune diseases. Therefore, further studies are warranted to uncover the specific role of redox signaling in MDSCs in autoimmunity and infection.

## Author contributions

KO and KT both researched data for the article, contributed to discussion of the content as well as writing and reviewing of the manuscript.

### Conflict of interest statement

The authors declare that the research was conducted in the absence of any commercial or financial relationships that could be construed as a potential conflict of interest.

## Abbreviations

ATRA: All-trans retinoic acid
CDDO-Me: C-28 methyl ester of 2-cyano-3, 12-dioxooleana-1, 9-dien-28-oic acid
Cys: cysteine
GSH: Glutathione
HCC: Hepatocellular carcinoma
HIF: Hypoxia-inducible factor
Keap1: Kelch ECH associating protein 1
MDSC: Myeloid-derived suppressor cell
M-MDSC: monocytic MDSC
NOX: NADPH oxidase
Nrf2: Nuclear factor (erythroid-derived 2)-like 2
OXPHOS: oxidative phosphorylation
PMN-MDSC: polymorphonuclear MDSC
PPP: pentose phosphate pathway
ROS: reactive oxygen species.
